# Deep Learning Glioma Grading with the Tumor Microenvironment Analysis Protocol for Comprehensive Learning, Discovering, and Quantifying Microenvironmental Features

**DOI:** 10.1007/s10278-024-01008-x

**Published:** 2024-02-27

**Authors:** M. Pytlarz, K. Wojnicki, P. Pilanc, B. Kaminska, A. Crimi

**Affiliations:** 1 Sano - Centre for Computational Personalised Medicine, Czarnowiejska 36, Kraków, 30-054 Poland; 2 Nencki Institute of Experimental Biology of the Polish Academy of Sciences, 3 Pasteur Street, Warszawa, 02-093 Poland

**Keywords:** Automated glioma grading, Deep learning, Quantification of tumor microenvironment elements, Tissue microarrays, Human leukocyte antigen

## Abstract

Gliomas are primary brain tumors that arise from neural stem cells, or glial precursors. Diagnosis of glioma is based on histological evaluation of pathological cell features and molecular markers. Gliomas are infiltrated by myeloid cells that accumulate preferentially in malignant tumors, and their abundance inversely correlates with survival, which is of interest for cancer immunotherapies. To avoid time-consuming and laborious manual examination of images, a deep learning approach for automatic multiclass classification of tumor grades was proposed. As an alternative way of investigating characteristics of brain tumor grades, we implemented a protocol for learning, discovering, and quantifying tumor microenvironment elements on our glioma dataset. Using only single-stained biopsies we derived characteristic differentiating tumor microenvironment phenotypic neighborhoods. The study was complicated by the small size of the available human leukocyte antigen stained on glioma tissue microarray dataset — 206 images of 5 classes — as well as imbalanced data distribution. This challenge was addressed by image augmentation for underrepresented classes. In practice, we considered two scenarios, a whole slide supervised learning classification, and an unsupervised cell-to-cell analysis looking for patterns of the microenvironment. In the supervised learning investigation, we evaluated 6 distinct model architectures. Experiments revealed that a DenseNet121 architecture surpasses the baseline’s accuracy by a significant margin of 9% for the test set, achieving a score of 69%, increasing accuracy in discerning challenging WHO grade 2 and 3 cases. All experiments have been carried out in a cross-validation manner. The tumor microenvironment analysis suggested an important role for myeloid cells and their accumulation in the context of characterizing glioma grades. Those promising approaches can be used as an additional diagnostic tool to improve assessment during intraoperative examination or subtyping tissues for treatment selection, potentially easing the workflow of pathologists and oncologists.

**Graphical Abstract:**

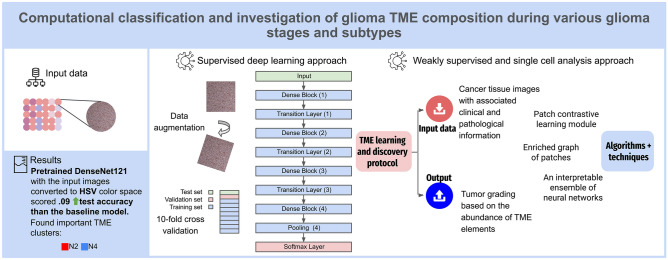

## Introduction

Glioma is a primary brain tumor that includes glioblastoma (GBM) as its most severe grade. It ranks among the deadliest tumors in adults. Despite treatment such as surgery, radiotherapy, and chemotherapy, survival rates for both men and women remain low [1]. Glioblastomas are heterogeneous and comprise tumoral, stromal, and immune cells that dynamically respond to many environmental stressors and therapies. In GBMs up to 30–50% of the tumor mass is composed of resident and infiltrating myeloid cells [2] that participate in creating a specific tumor microenvironment (TME) supporting tumor progression. The current World Health Organization (WHO) classification criteria for gliomas combine histological and genomic indicators [3]. WHO defined three main groups of adult-type diffuse gliomas — Astrocytoma IDH-mutant, Oligodendroglioma IDH-mutant, and Glioblastoma IDH-wildtype — classified according to four histopathological grades [4] depending on tumor malignancy, nuclear atypia, and degree of invasion of the surrounding brain parenchyma. The microenvironment of the tumor is crucial to its progression, which can be delayed with immunotherapy, exploring various immunotools or viruses to kill tumor cells or reinvigorate antitumor immunity. Clinical trials are currently ongoing to evaluate the safety and efficacy of an arsenal of immunotherapeutics for the treatment of newly diagnosed and recurrent high-grade gliomas. The challenge is to identify which patient populations will benefit the most from these medications, and why [5]. Differences in immune cell proportions and phenotypes within tumors appear to be determined by molecular characteristics of glioma cells [6–8]. Growing evidence highlights the significance of interactions between myeloid cells and glioma cells, enabling them to evolve in a way that supports tumor growth. Spatial interactions between tumor and immune cells have been linked to clinical responses to immunotherapies in GBM patients [9].

Even if invasive, histopathology continues to be the primary method for determining the diagnosis and prognosis of cancer. In practice, a neuropathologist would investigate stained tissue slices under a microscope to diagnose and classify brain tumors. This has led to extensive research on artificial intelligence for digital pathology [10] exploring image features along with the corresponding quantitative classification, integrating different types of clinical and molecular data for improved prognosis accuracy. Mobadersany et al. [11] accurately forecast cancer outcomes based on histology and genomics data utilizing deep learning techniques, which include convolutional layers trained to predict image patterns associated with survival. In another study, a deep learning architecture was used to create maps of tumor-infiltrating lymphocytes [12] through segmenting hematoxylin and eosin ($H \& E$) stained images from The Cancer Genome Atlas archive.

Tumor subtyping and grading are of great interest for research on various types of cancer [13]. In this study, we describe a similar approach but focus on histological images with human leukocyte antigen (HLA) staining, highlighting immune components. We consider supervised deep learning, weakly supervised deep learning (WSDL), and single-cell analysis (SCA). While WSDL extracts essential histopathological tumor features blindly from volumes of raw or weakly annotated images of stained tissue sections, the latter method is more focused on quantifying morphology elements of the microenvironment present in patches of the image, and requires more advanced and time-consuming analysis. WSDL assigns labels during training, while SCA relies on the structure and patterns within the data itself. It is important to note that typical SCA methods alone are sequential and not learning-based. This means that the extracted phenotypes and neighborhoods are inferred without consideration of the specific clinical questions on hand. Consequently, the quantified microenvironmental features may not be optimal for differentiating between patient types. Complementing SCA, WSDL employs various types of convolutional neural networks to predict patient-level labels.

The purpose of this study is to evaluate the hypothesis that automatic grading and subtyping from single-stained histological slides can constitute an intraoperative step during surgery, as well as in other forms of non-invasive therapy planning [14]. Moreover, researchers anticipate that neuropathologists and machine learning models will make different sorts of categorization errors and that the aggregate assessment of a hybrid pathologist/machine learning model will be superior to either human or machine assessment alone [15].

### Tissue Microarrays

Data used for the model training included digital images of tissue samples stained to reveal human leukocyte antigen HLA-DP, -DQ, -DR on human glioma tissue microarray (TMA) [16]. HLA antibody recognizes a major histocompatibility complex (MHC) class II heterodimer cell surface receptor expressed primarily on antigen-presenting cells such as B lymphocytes, monocytes, and macrophages. MHC class II molecules bind intracellularly processed peptides and present them to T-helper cells [17]. Sample preparation is described in the next session. HLA staining reveals predominantly glioma-infiltrating myeloid cells that accumulate in the tumor and have been demonstrated to play an important role in glioblastoma biology and can be used as a diagnostic biomarker [18]. Fan et al. showed an increased HLA-DR score in more aggressive stages of glioma. The authors state that HLA-DR can be used to predict the tumor grade in gliomas [19]. The choice of TMAs is justified by the fact that commercial TMAs constitute multiple, reproducible, and histologically well-verified sections of tumor and normal brain tissue. The tissue microarray technique enables highly detailed immunohistochemical characterization of huge numbers of cancer cases. The use of antibodies can clearly illustrate the heterogeneity of malignant tumors [20]. Tissue sections obtained from TMA can be subjected to a vast array of regular stains, special stains, immunostainings, and morphology-based molecular methods with remarkably high-quality results [21]. TMAs are more reproducible than tumor samples or independently collected tumor sections, especially when obtained from separate laboratories. Less reproducible sections would have an impact on immunocytochemical staining and make multisection comparisons more difficult. Because TMAs are employed by so many researchers, histopathological examination is performed by numerous pathologists, resulting in a more trustworthy evaluation. Obtaining several normal brain sections for comparison is usually challenging, but in the case of TMA, such sections are available.

### Supervised Deep Learning

Convolutional neural networks (CNNs) have been successfully used to classify histology slides, mostly breast parenchyma [22–26] possibly due to the availability of breast specimen histology datasets and the relatively high incidence of breast tumors compared to those of other organs. However, there are studies in which authors use transfer learning to apply weights learned on one organ’s histology dataset to another organ’s dataset [27]. Ertosun and Rubin used a combination of two CNNs to categorize histology slides of glioma brain tumors into distinct tumor grades [28]. In 2022 Inception V3 was identified as the best-performing model [29] in image recognition methods across five public histopathology datasets. While ResNet [3] and Inception V3 represent modern CNNs, vision transformers (ViTs) are beginning to challenge them in computer vision for natural images. Chen et al. [30] implemented a ViT leveraging the natural hierarchical structure inherent in whole-slide images, and it outperformed other models for cancer subtyping. However, transformer models lack the translation equivariance and locality of CNNs; as a result, vision transformers are less effective with limited datasets. In addition to requiring significant computing power (especially compared to CNNs) due to their self-attention mechanism, they are also memory-intensive, have a large number of parameters, and exhibit limited generalization capabilities on small datasets.

### Weakly Supervised Deep Learning and Single-Cell Analysis

In this study, we investigate two approaches to automating the analysis of tumor histopathology or phenotype: WSDL and SCA. WSDL automatically infers fine-grained (pixel- or patch-level) information from coarse-grained (image-level) annotations. WSDL significantly reduces pathologists’ annotation workload [31, 32]. On the other hand, SCA segments cells in the tissue, measures their shape and marker expression intensity, and identifies clusters with similar characteristics, as well as higher-order interactions or phenotypic “neighborhoods” [33]. In other words, the data obtained from SCA cell segmentation and marker expression intensity measurement [34] is used to recognize phenotypic neighborhoods. SCA techniques accomplish this by constructing topological networks with cell phenotypic interactions and using graph-based clustering to allocate groups of cells to distinct neighborhoods. SCA approaches have a high level of interpretability since they use the cell as the main unit of tissue depiction [35]. The cell-to-graph approach extracts individual cells from the images and represents their spatial relationships as graphs allowing tracking of the intricate cellular interactions within tumors. In tumor grading, the cell-to-graph approach can be used to automatically quantify various morphological features of cells, such as size, shape, and arrangement, and classify tumors into distinct grades. By representing cells as nodes and their spatial relationships as edges in a graph, the cell-to-graph approach enables analysis of cellular interactions, such as cell clustering, spatial distribution, and connectivity patterns, which can yield valuable insight regarding the behavior and prognosis of tumors. This approach can help automate and standardize tumor grading, reduce subjectivity and inter-observer variability, and provide quantitative and reproducible measures for tumor characterization, which, in turn, aids clinical decision-making.

Zhou and colleagues [36] created a cell graph convolutional network utilizing nuclear appearance features and spatial location of nodes to enhance its performance. The cell graph convolutional network also introduces Adaptive GraphSage, a graph convolution technique that combines multi-level features in a data-driven manner, to enable nodes to fuse multiscale information. To address redundancy in the graph, a sampling technique is proposed to remove nodes in areas of dense nuclear activity. The cell graph convolutional network approach can capture the complicated tissue microenvironment by considering images 16 times larger than patch-based methods. Pati et al. [37] introduced a novel multilevel hierarchical entity-graph representation of tissue specimens for cancer diagnosis and prognosis. Traditional approaches have encoded cell morphology and organization using cell graphs, but this study proposes a hierarchical graph representation that captures histological entities at multiple levels, from fine to coarse. The study demonstrates the potential of using multi-level hierarchical entity-graph representations for encoding tissue structure and function in cancer diagnosis and prognosis, and it is evaluated on a large cohort of breast tumor specimens. Lastly, there has been work on explainable graphs in digital pathology. This refers to graph-based techniques that are employed to represent and assess digital pathology images in a way that provides more control over concept representation and allows for comprehensive and compact explanations. Jaume et al. [38] introduced a post-hoc explainer that derives compact per-instance explanations emphasizing diagnostically important entities in the graph. They prune the redundant and uninformative graph components, and the resulting sub-graph is defined as the explanation. Yu et al. [39] proposed a method called IFEXPLAINER, which generates necessary and sufficient explanations for graph neural networks. The explanations are based on the measurement of information flow from different input subgraphs to the predictive output of the network. The authors introduced a novel notion of predictiveness called *f*-information, which incorporates the realistic capacity of the graph neural network model, and uses it to generate the explanatory subgraph with maximal information flow to the prediction. The produced explanation is essential for prediction and sufficient to reveal the most crucial substructures.

Taking into consideration the lessons learned and limitations of the aforementioned approaches, we investigated the two scenarios of whole slide classification and single-cell analysis, which are described in the following sections.

## Materials and Methods

### Data

**Fig. 1:**
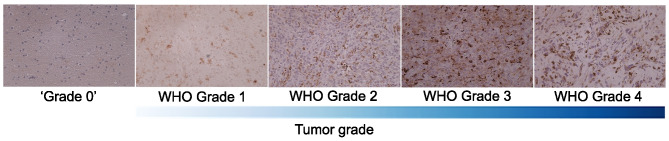
Examples of Human Leukocyte Antigen (HLA)-stained tissue microarray’s cores. The chosen examples have clear histological differences. However, in some cases, differences in the highest grade are difficult to grasp with the naked eye

The tissue microarrays used in the experiments are complex, commercially available paraffin blocks constructed by extracting cylindrical core tissue “biopsies” from different donor paraffin blocks and embedding them again in a single recipient block (microarray) at specific matrix coordinates [21]. Of those TMA were sections representing 21 normal brain tissue samples, 24 samples of WHO grade 1, 84 grade 2, 26 grade 3, and 51 grade 4 gliomas (Fig. 1). 63$\%$ of malignant samples were collected from male patients and 37$\%$ of malignant samples — from female patients. For the sake of convenience and consistency, we named normal tissue samples “grade 0.”

### HLA Immunostaining

We have used commercial GL2083b and GL806g TMAs from TissueArray.LLC.com (MD, USA) containing multiple GBM cases, and normal brain tissues with duplicate cores per case. Paraffin-embedded sections were incubated for 10 min at 60 °C, deparaffinized in xylene, rehydrated in ethanol (100%, 90%, 70%), and washed with water. Epitopes were retrieved by boiling in a pH 6.0 citrate buffer for 30 min. Further steps (blocking of endogenous peroxidase and unspecific interactions, application of secondary antibodies, and signal development) were performed using reagents from the ImmPRESS^®^ kit according to the manufacturer’s protocol. Primary antibody recognizing HLA-DP, -DQ, -DR antigen (anti-HLA-DP, -DQ, -DR, Dako, #M0775) diluted in 3% donkey serum was applied for overnight incubation in 4${}^{\circ }\text {C}$. All washes were performed using PBS-T (Tween-20, 0.05%). Sections were counterstained with hematoxylin washed in H$_2$O, dehydrated, and mounted using a glycerol mounting medium. Images were acquired with the Leica DM4000 B microscope operating with the Application Suite ver. 2.8.1 software (Leica Microsystems CMS, Switzerland).

### Supervised Deep Learning Approach

Small sample size and imbalanced data distribution posed two main challenges for the subsequent multiclass classification task; as images of WHO grade 2 tumors were approximately three times overrepresented in comparison to other grades. To address the issue of the imbalanced dataset and check if the model’s performance will improve, we split in a stratified 10-fold cross-validation style for the validation, training, and test subsets, and then augment histologies of normal brain tissue and WHO grades 1, 3, and 4 by rotating the view of 90 and 180°. As most of the deep learning architecture is not rotation invariant, rotated images can be considered as separated samples [40].

During preprocessing, the tissue microarray well views were center-cropped and the size was adjusted from 19,200 × 1200 pixels to 1200 × 1200 pixels. To investigate the impact of preprocessing our input images, we conducted experiments by converting the images from the standard RGB color space to the HSV (hue, saturation, value) color space. Converting input images to HSV may enhance the model’s ability to discern patterns by separating color from intensity.

We chose to use a CNN architecture not susceptible to vanishing or exploding gradients, which is addressed by a deep residual learning framework [41]. In this study, the baseline model implemented was the simplest among ResNets–ResNet18, with a 7 × 7 convolutional layer filter, then 16 convolutional layers with a 3 × 3 size filter and average pooling. Output was passed through the SoftMax activation function. The pretrained model with only the last layer being updated was trained within 10 iterations for different data shuffles to split subsets and augmentation. Data augmentation was performed on the training dataset, avoiding redundancy in the validation set. We employed the cross-entropy criterion as the loss function. Choosing a moderately complex model as a baseline allows for a balance between model capacity and computational efficiency. ResNet18 is a relatively lightweight deep learning model compared to deeper architectures like ResNet50. ResNet architectures, in general, are considered a community standard and are often used as a reference point in research papers [42–46]. ResNet18 has been chosen as a more resource-efficient option (compared to DenseNet121 or VGG16). The ultimate goal is to compare different architectures, and ResNet18 pretrained serves as a reference point for this comparison. The concentration is on understanding the relative performance of each model. We conducted a set of experiments to explore the impact of various neural network architectures on image classification on a small dataset. Our primary focus was on comparing these architectures to the ResNet18 pretrained baseline, considering three key aspects of comparison: (1) parameter efficiency, (2) depth, and (3) architectural representative features. We selected five popular neural network architectures known for their effectiveness in computer vision: DenseNet121 [47], EfficientNet [48], MobileNetV3 Small [49], VGG16 [50], and ResNet50 [41]. Two main experimental setups were employed to understand the performance of these architectures. Firstly, all selected architectures were trained from scratch, providing insights into their ability to learn representations from the given dataset. Secondly, we utilized transfer learning by applying a pretrained model and updating only the last layer’s weights. Finally, we tested all the models trained from scratch and pretrained both with prior basic image preprocessing and converting input images to the HSV color space. For a nuanced understanding of the architecture search models, we discuss three key aspects of comparison mentioned. (1) Parameter efficiency: we specifically tested MobileNetV3 Small and EfficientNet-B0, designed for parameter efficiency (number of trainable parameters of the models applied is listed in Table 1. These architectures are tailored for resource-constrained environments and in our small dataset case these networks could prove to be beneficial because of not having too many parameters potentially leading to overfitting. (2) Depth: ResNet50 and DenseNet121, chosen for their depth, are capable of handling complex tasks. VGG16 was picked to test if, in contrast, its simplicity and uniform structure could work better for our task and small dataset. (3) Architectural representative features: each architecture represents unique features. DenseNet introduces dense connectivity, fostering feature reuse. EfficientNet and MobileNetV3 optimize the balance between model size and performance. ResNet contains residual connections, addressing training challenges associated with deep networks.

**Table 1:** Metrics averaged from 10 folds of each model assessing their performance for comparison. Rows arranged in order of validation loss — from lowest to highest. Bolded are the values of the baseline model - ResNet18 pretrained - and DenseNet121 pretrained HSV, with an accuracy of 69% on the test set significantly higher compared to the baseline’s 60% test accuracy

*Model ID*	*Num of trainable params*	*Avg fold runtime (min)*	*Train accuracy*	*Val accuracy*	*Val F1*	*Val loss*	*Test accuracy*	*Test F1*	*Test loss*
*ResNet50 pretrained HSV*	10245	4.16	.75±.04	.64±.1	.53±.14	.93±.13	.64±.07	.56±.1	.94±.12
***DenseNet121 pretrained HSV***	**5125**	**6**.**5683**	**.73±.02**	**.69±.09**	**.59±.13**	**.95±.11**	**.69±.06**	**.61±.08**	**.93±.07**
*EfficientNet pretrained HSV*	6410	4.5983	.75±.03	.66±.09	.59±.11	.95±.13	.65±.06	.59±.1	.95±.16
*ResNet50 pretrained*	10245	3.305	.73±.03	.65±.11	.57±.07	.97±.15	.61±.1	.55±.08	.99±.11
*DenseNet121 pretrained*	5125	5.025	.75±.02	.64±.08	.58±.07	.98±.11	.65±.1	.57±.13	.94±.12
*ResNet18 pretrained HSV*	2565	3.405	.68±.03	.65±.07	.56±.09	1.0±.12	.65±.06	.54±.11	1.0±.11
***ResNet18 pretrained***	**2565**	**2**.**985**	**.72±.02**	**.65±.09**	**.55±.12**	**1.01±.11**	**.6±.11**	**.5±.15**	**1.03±.13**
*EfficientNet pretrained*	6410	2.915	.75±.02	.58±.09	.51±.07	1.07±.11	.62±.11	.54±.13	1.02±.16
*MobileNet HSV*	1522981	1.9167	.86±.08	.58±.05	.54±.07	1.45±.32	.58±.13	.5±.12	1.36±.59
*DenseNet121 HSV*	6958981	1.5283	.88±.02	.58±.06	.5±.08	1.52±.25	.62±.12	.53±.13	1.16±.23
*DenseNet121*	6958981	1.12	.85±.05	.53±.15	.49±.18	1.54±.57	.57±.1	.49±.12	1.19±.38
*VGG*	134281029	6.4426	.67±.09	.52±.12	.39±.11	1.55±.75	.5±.16	.38±.17	1.27±.28
*VGG HSV*	134281029	7.2815	.68±.11	.49±.13	.43±.12	1.64±.46	.55±.06	.34±.09	1.29±.18
*MobileNet*	1522981	1.455	.89±.06	.52±.11	.47±.15	1.72±.77	.56±.09	.48±.16	1.33±.35
*MobileNet pretrained HSV*	1522981	1.5033	1.0±.01	.67±.09	.62±.13	1.82±.7	.61±.1	.57±.11	1.35±.32
*ResNet18 HSV*	11179077	2.1633	.97±.04	.51±.09	.43±.05	1.88±.85	.56±.08	.48±.09	1.27±.34
*ResNet18*	11179077	1.41	.97±.02	.49±.07	.44±.1	1.91±.27	.55±.1	.47±.12	1.26±.3
*EfficientNet HSV*	4013953	3.36	.88±.11	.51±.12	.45±.14	1.96±.75	.53±.11	.46±.15	3.18±5.52
*ResNet50 HSV*	23518277	3.4083	.81±.05	.47±.07	.4±.07	1.98±.48	.5±.14	.42±.17	1.29±.4
*EfficientNet*	4013953	2.71	.87±.1	.53±.08	.48±.1	2.28±1.27	.51±.17	.43±.18	1.63±1.13
*VGG pretrained*	134281029	3.8463	.9±.04	.5±.13	.47±.16	2.38±1.18	.56±.12	.51±.16	1.15±.24
*ResNet50*	23518277	2.9983	.82±.07	.38±.18	.35±.21	2.43±.7	.56±.08	.47±.09	1.28±.31
*VGG pretrained HSV*	134281029	4.7074	.91±.06	.51±.08	.46±.07	2.63±.87	.58±.16	.48±.2	1.14±.3
*MobileNet pretrained*	1522981	1.2067	.99±.01	.56±.1	.52±.08	2.72±.68	.58±.09	.52±.11	1.68±.65

### Single-Cell Analysis and Weakly Supervised Deep Learning

Supervised deep learning, a foundational technique, involves training a model on labeled data, where input-output pairs guide the network in learning patterns and making predictions. Conversely, weakly supervised deep learning relies on partially labeled or noisy data, providing broader flexibility by requiring less annotation effort. In the context of self-supervised learning, a subset of unsupervised learning, the emphasis shifts from clustering and grouping to regression and classification tasks.

In the second part of our study, we expanded a previously introduced pipeline for cancer tissue analysis [51] with our objective of quantifying the glioma microenvironment. The approach integrates cell-level interpretable quantification of the TME with patch-based weakly supervised learning of tumor histopathology to automate the in situ discovery of clinically relevant TME elements. The learning and quantifying protocol is a multi-level, interpretable deep learning ensemble that discovers the most important TMEs from tissue sections while performing a classification task using only patient-level labels. The algorithm assigns patches to TMEs at three levels of spatial complexity: local cell phenotypes, cellular neighborhoods, and tissue area-specific interactions between neighborhoods.

The protocol was initially designed for retrieving computationally specific cancer biomarkers from multiplexed stained tissues — here, we applied the learning and quantifying algorithm to search if a useful portion of interactions and abundances of tumor features along grades of malignancy could be retrieved. The input data consists of images of HLA-DR-stained (single stain) glioma TMAs with clinical and pathological data on their WHO grades.

Patch contrastive learning (PCL) is a technique within self-supervised learning, which encodes high-dimensional pixel information into 256-dimensional vectors of enriched patch embeddings. PCL operates iteratively, training on randomly selected patches through an unsupervised approach. A contrastive loss function maximizes agreement between augmented views of closely located patches while minimizing agreement between augmented views of distant patches. In other words, it involves breaking down images into smaller patches and training a model to distinguish between similar and dissimilar patches. The integration of PCL into our framework becomes a cornerstone in extracting clinically relevant insights from complex biological data and comprehensive modeling of the tumor microenvironment (TME).

After the graph of patches containing the spatial interactions between tissue patches is generated, it is passed to the interpretable ensemble of neural networks. These neural networks learn phenotypes, phenotype neighborhoods, and areas of interaction between neighborhoods to classify patients based on the abundance of TMEs.

Implementation discovers tumor landscape characteristics that are relevant to a certain predictive task. This can be done a posteriori by analyzing the TMEs obtained by the ensemble of networks while classifying patients. It is the purpose of the BioInsights module, which is accomplished by identifying cohort-differentiating features (differential TME analysis) and significant TMEs in individual predictions. The continuous flow from PCL to patient classification forms the backbone of tumor microenvironment investigation protocol, facilitating a grasp of intricate relationships within cancer tissue images.

To sum up, these are the main steps of the pipeline described above:

1. **Input data:** Cancer tissue images with associated clinical and pathological information.
2. **Patch contrastive learning module:**
  **Image division:** The module systematically divides images into patches, breaking down the data into per-cell units.
  **Vector embedding:** Each patch transforms in a 256-dimensional vector using a CNN.
  **Biological structure recognition:** The CNN is trained in an unsupervised manner to assign similar vectors to patches containing similar biological structures.
3. **Enriched graph of patches:** This stage involves capturing the spatial interactions between tissue patches, creating an enriched graph.
4. **Interpretable ensemble of neural networks:**
  **Phenotype learning:** The ensemble of networks learns phenotypes from the enriched graph of patches.
  **Neighborhood analysis:** The model identifies phenotype neighborhoods and areas of interaction between these neighborhoods.
  **Patient classification:** Based on the abundance of tumor microenvironment elements, the ensemble of networks classifies patients.

Codes of the methods described above are provided: https://github.com/octpsmon/TME_analysis_protocol_n_glioma_grading.

## Results

### Results from Supervised Deep Learning Classification

**Fig. 2:**
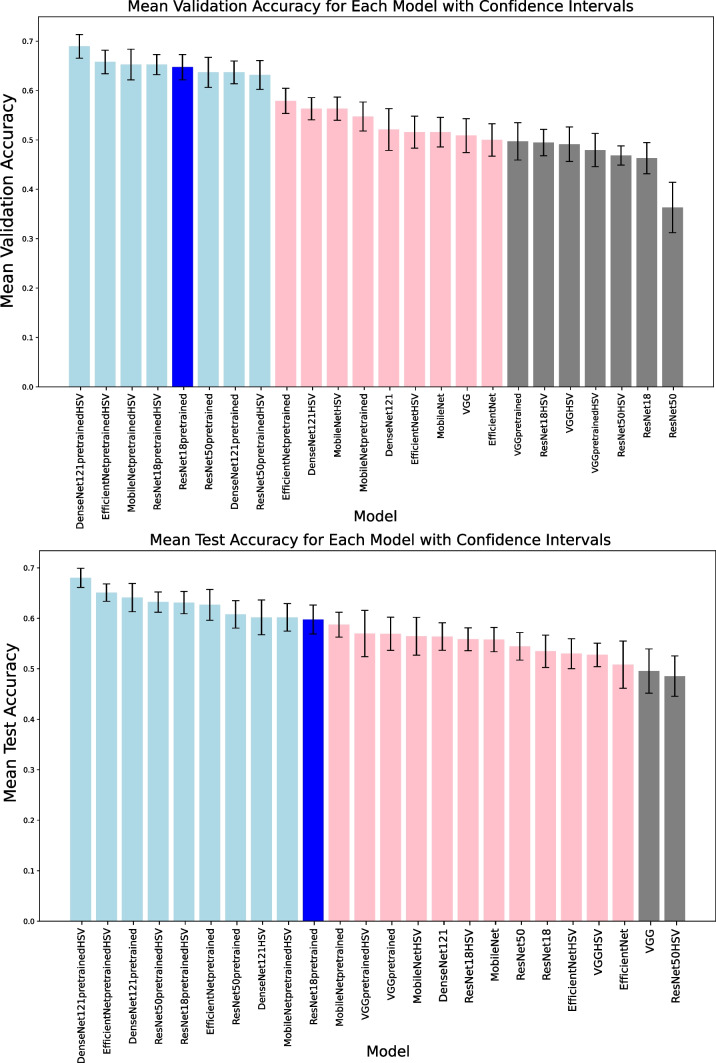
Performance averaged across 10 folds of each model. The “HSV” suffix indicates the model tested on input images in HSV color space. Scores not reaching 50% were marked in gray, between 50 and 60% in light red, and those exceeding 60% in light blue

An examination of the results derived from 24 supervised learning experiments has been conducted (Table 1, Fig. 2). The benchmark for these evaluations — ResNet18 pretrained model — attained 65% validation accuracy, 60% test accuracy, and a validation loss of 1.01. Models exhibiting superior performance in terms of validation accuracy include: ResNet18 pretrained HSV (“HSV” suffix in this context means incorporating additional preprocessing involving the conversion of input images to the HSV color space), MobileNetV3 small pretrained HSV, EfficientNet ptretrained HSV, and DenseNet121 pretrained HSV. Concurrently, models surpassing the baseline in terms of test accuracy encompass DenseNet121 HSV, ResNet50 pretrained, EfficientNet pretrained, ResNet18 pretrained HSV, ResNet50 pretrained HSV, DenseNet121 pretrained, EfficientNet pretrained HSV, and DenseNet121 pretrained HSV. Interestingly, all the experiments with better performance on the validation set and 5 of 9 experiments with better results than the baseline on the test set include a preprocessing step of converting input images to the HSV color space. Among the best scoring models that were applied as pretrained there is one trained from scratch better than the baseline on the test set — DenseNet121 HSV with almost 7M of trainable parameters.

Beyond mere absolute value comparisons, we systematically evaluated the statistical significance of the observed differences by subjecting each model to a comparison with the baseline. To this end, we employed the Mann-Whitney *U* test, a non-parametric method designed for comparing two independent groups when the data distribution is non-normal. The rationale for selecting this test stems from the nature of our experimental setup, wherein 24 deep-learning models were individually trained and tested on the same dataset, employing 10 distinct splits for training, validation, and testing. This design ensures the independence of the data, as each model’s performance is appraised on a unique set, despite originating from the same dataset. Shapiro-Wilk test results decisively revealed that both validation and test accuracies do not conform to a normal distribution. Mann-Whitney *U* test reveals that DenseNet121 pretrained HSV model demonstrated a statistically significant superiority over the ResNet18 pretrained model in terms of test accuracy, as evidenced by a U-statistic of 27.000, a *p*-value of 0.029, and a median difference of 0.067. Therefore DenseNet121 pretrained HSV model’s accuracy of 69% on the test set is significantly higher compared to the baseline’s 60% test accuracy.

**Fig. 3:**
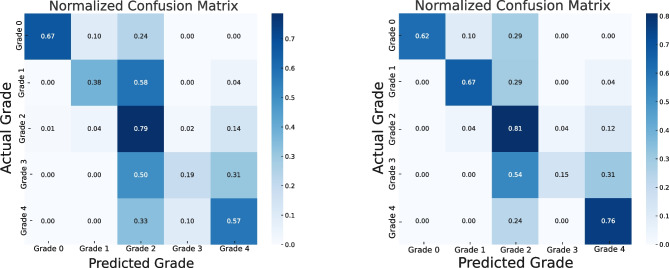
Confusion matrices. On the left: ResNet18 pretrained evaluation on the test set. On the right: DenseNet121 pretrained with HSV color space input evaluation on the test set

We scrutinized the performance of the baseline vs DenseNet121 pretrained HSV through the lens of confusion matrices derived from predictions on the test set (Fig. 3). Both models recognize normal brain tissue with the same accuracy. DenseNet has generally slightly higher accuracy in WHO grades 1, 2, 3, and 4. DenseNet tends to appear better at correctly identifying positive cases and not misclassifying negative cases as positive. The F1 score, balancing precision and recall, is generally higher for DenseNet across most grades.

### Results from Weakly Supervised Deep Learning and Single-Cell Analysis

Results from contrastive learning (Table 2) reveal the model has strong discriminatory power (Area Under the Receiver Operating Characteristic curve — AUC-ROC — of range 0, 8–0, 9) to differentiate between grades 0 and WHO grade 1, as well as WHO grades 1 and 4. The model obtained good results (AUC-ROC of 0, 7–0, 8) when distinguishing between the following grades: (0, 4), (1, 2), (1, 3). An acceptable score (AUC-ROC of 0, 6–0, 7) was shown in the discrimination between grades: (0, 2), (0, 3). Patch contrastive learning is not enough reliable when it comes to differentiating grades: (2, 3), (2, 4), (3, 4).

**Table 2:** Accuracy, sensitivity, specificity, and ROC curve area scores in tumor grade classification across 10 binary comparisons

*Binary classification — tumor grades*	*0, 1*	*0, 2*	*0, 3*	*0, 4*	*1, 2*	*1, 3*	*1, 4*	*2, 3*	*2, 4*	*3, 4*
*Accuracy*	73%	55%	68%	78%	79%	70%	79%	63%	48%	51%
*Sensitivity*	86%	95%	71%	86%	54%	75%	75%	68%	76%	81%
*Specificity*	63%	45%	65%	74%	86%	65%	80%	46%	32%	35%
*ROC curve area*	87%	65%	69%	75%	79%	75%	88%	58%	52%	56%

**Fig. 4:**
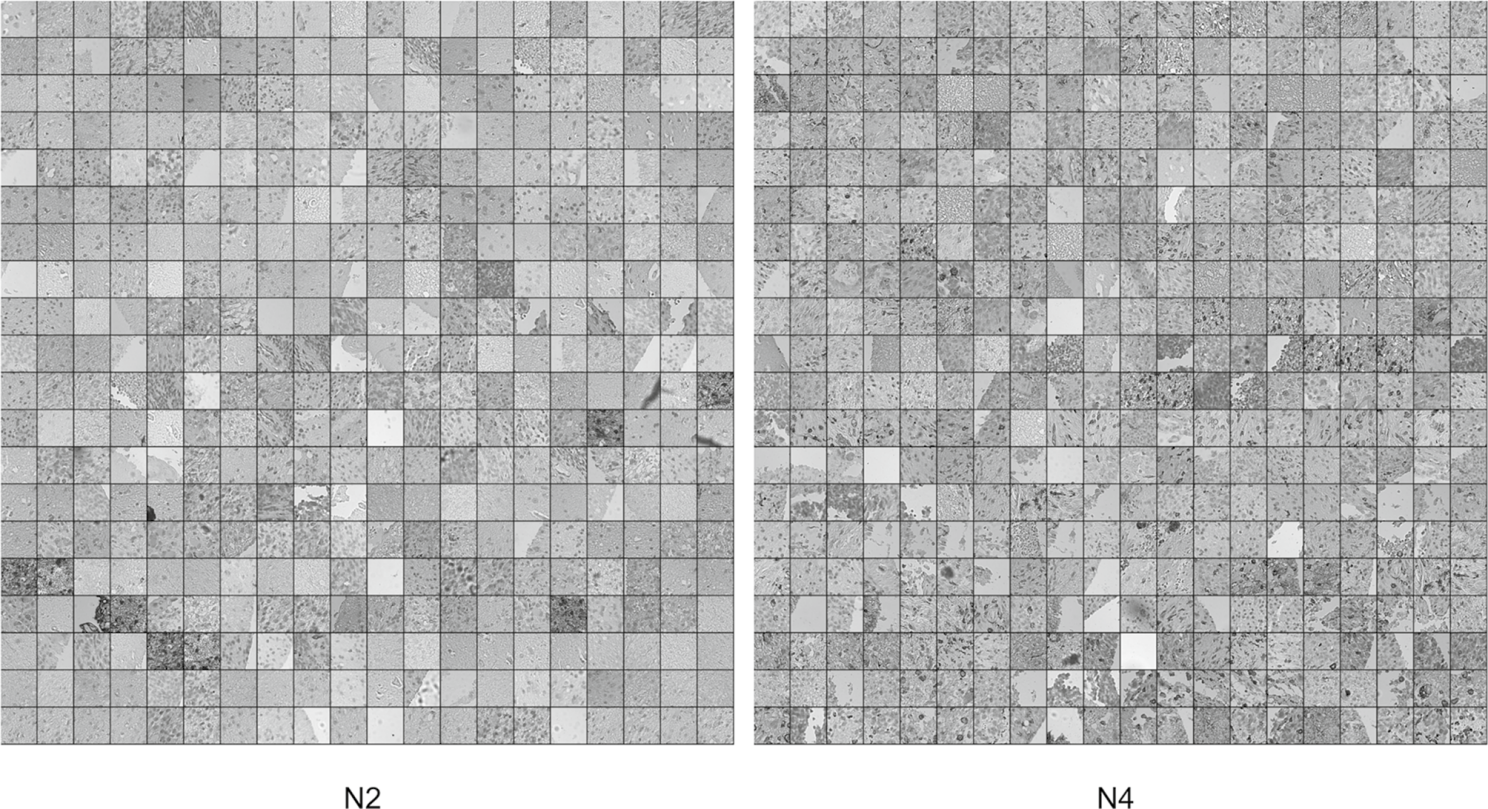
View of clusters (neighborhoods). This figure presents 2 frames made of patches of the tumor images that were assigned together within 2 groups during patch contrastive learning protocol and resulted in the 2 most distinctive clusters of cell neighborhoods

The learning and quantifying algorithm learned to recognize 23 patterns of TME elements — 8 phenotypes, 8 cell neighborhoods, and 7 areas of neighborhoods. The quantitative model distinguishes the highest number of differences between grade 0 and WHO grade 4. It also recognizes a high significantly increased abundance of cells of neighborhoods N2 in WHO grade 4 compared to WHO grade 1 as well as a minimally significantly increased abundance of the neighborhoods N4, N6, N8, and N9 of cells and phenotypes P3, and P4 comparing these two. Neighborhood N2 detected in WHO grade 2 glioma is less significantly increased compared to grade 0 than in WHO grade 4 compared to 0. Therefore, N2 is highly predictive for patients with tumors of WHO grade 4. The neighborhood N4 appears significantly more frequently in WHO grade 3 compared to grade 0, suggesting that N4 was relevant to successfully classifying WHO grade 3. The neighborhood N3 is visible significantly more often in WHO grade 4 compared to WHO grade 2 (Fig. 4).

**Fig. 5:**
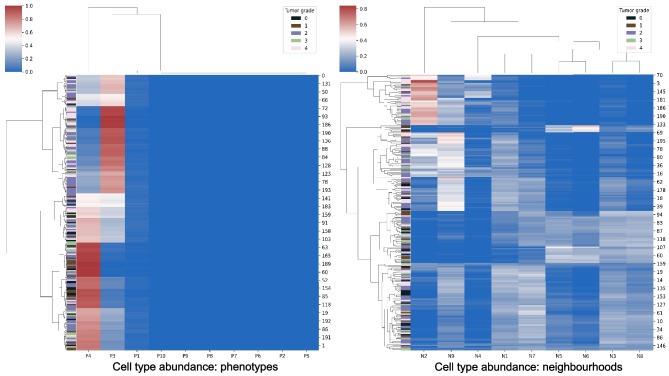
Cell type abundance. TME composition of fields — a heatmap showing the sum-to-1 normalized distribution of neighborhoods in 206 images/patients, colored by label (tumor grade). Both images/patients and neighborhoods are ordered by hierarchical clustering. The most abundant phenotypes are P4 and P3. The most abundant neighborhood is N2 — mostly in grade 4 and 2

**Fig. 6:**
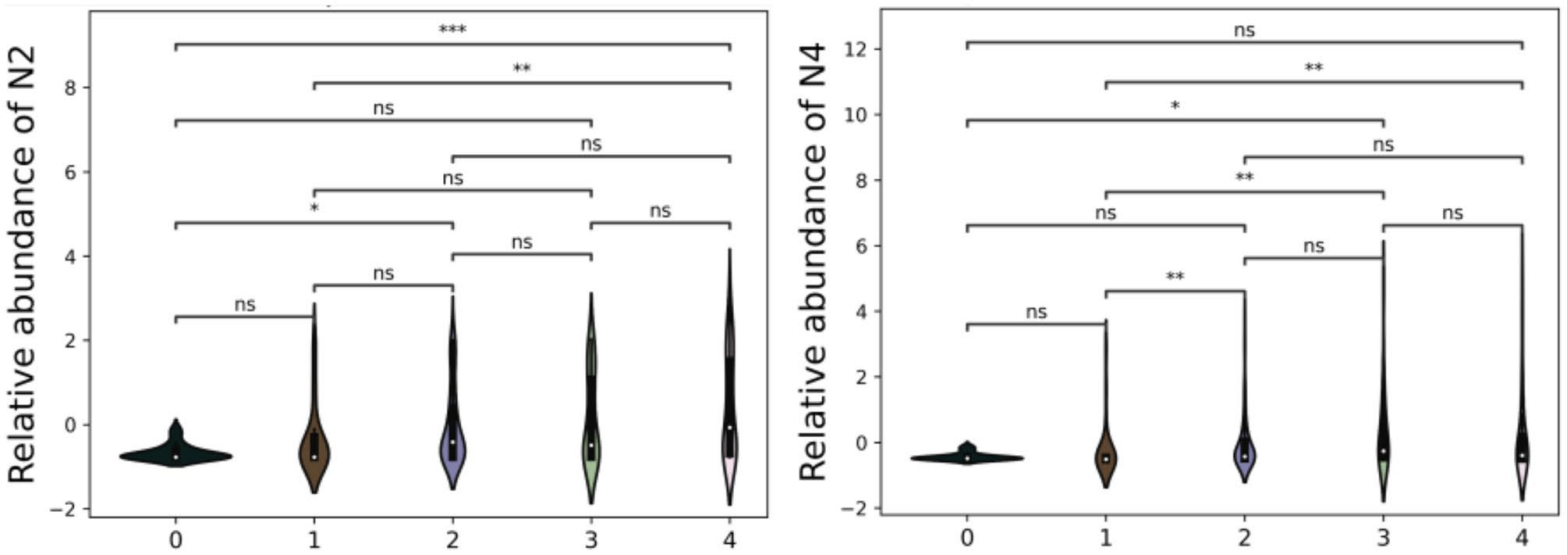
Violin plots showing a relative abundance of clusters. The plots represent how significant are particular differences between cluster abundance between tumor grades. The distribution of each plot illustrates the variation and density of the cluster abundance, emphasizing the statistical significance of group differences. Marking of *** indicates *p*-value of<0.001, marking of ** the *p*-value of<0.01, and marking of * the *p*-value of<0.05

**Fig. 7:**
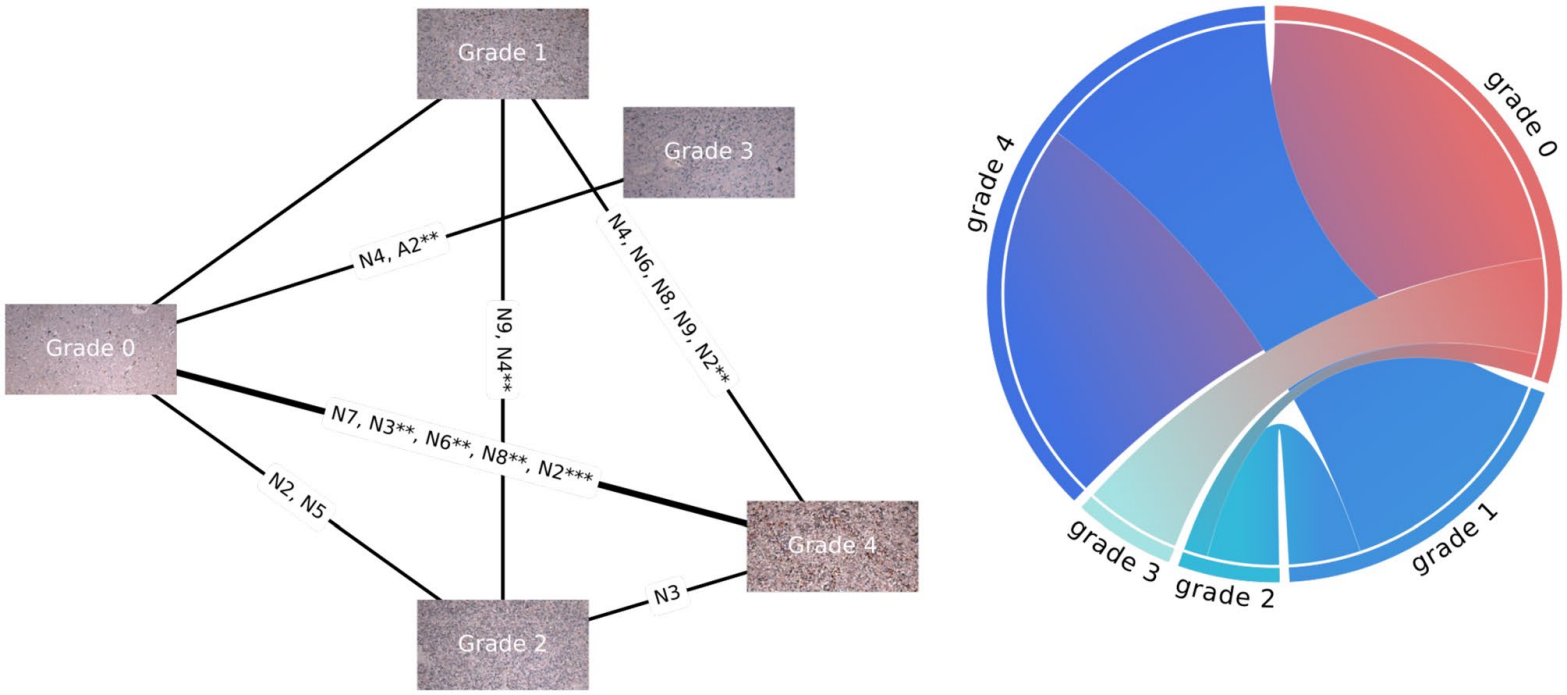
Graph of clusters of tumor microenvironment features. On the left: the undirected graph with connections linking tumors of various grades where occurred significant differences between cluster abundances. The bold line indicates two grades with the most different characteristics. On the right: a weighted sum of common clusters occurring with significant differences in the abundance between tumors of different grades. The diagram on the right is based on the same results as the graph on the left, but it more strongly represents the number and significance of the differences between grades (when the *p*-value is<0.01 the number of shared clusters is multiplied by 2, and when the *p*-value is<0.001 — the number of shared clusters is multiplied by 3)

N2 occurs most frequently on TMA core images (Fig. 5) and its abundancy has the highest significance when comparing tumor grade 0 with WHO grade 4, the significance of *p*-value less than 0.01 when comparing WHO grades 1 with 4, and significance of *p*-value less than 0.05 when comparing grade 0 with WHO grade 2. N9, the second neighborhood of phenotypes is associated with tumor WHO grades 1, 2, and 4 (Figs. 6 and 7). On the relative abundance plots, we can observe in detail how the neighborhoods N2 and N4 are the two showing the most significant differences between grades (Fig. 6). Figure 4 presents the actual view of the patches assigned to clusters N2 and N4. P3 and P4 are the phenotypes that appear most frequently in images of high-grade gliomas. The N4 neighborhood’s view might reflect an accumulation of protumor macrophages, resulting in increased HLA staining. The extracted characteristics include round and ameboid cells.

The global differential TME analysis revealed that the highest predictive influence has elements: N1, N2, N5, N8, and N9, which corresponds with measurements of the significance of their abundance in 206 images of different tumor grades. The higher the predictive influence ratio value, the higher the relevance of the TME element for a specific patient.

Violin plots (Fig. 6) present significant differences between derived clusters between tumor grades. N2 neighborhood occurs highly significantly more abundantly in the most malignant tumors than in tumor-adjacent tissue and moderately richer in the WHO grade 4 rather than in 1. While N4 neighborhood appears significantly more frequently in grade 4 than in grade 1 tumors, but also WHO grade 3 compared to 1. There is significance observed when comparing the relative abundance of cluster N4 between WHO grade 3 and normal brain tissue.

### Visualization with UMAP

**Fig. 8:**
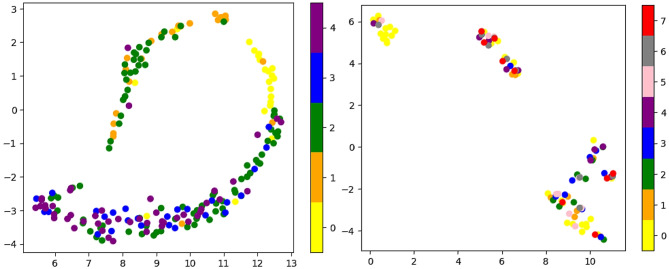
Left: UMAPs of tumor grades. UMAP plots with color-coded labels for tumor grades and neighborhoods. Starting from the left: 0, represents tissue “grade 0”; 1, tumor WHO grade 1; 2, tumor WHO grade 2; 3, tumor WHO grade 3; and 4, tumor WHO grade 4. Right: UMAPs of neighborhoods: 0, denotes N1 neighborhood; 1, N2 neighborhood; 2, N3 neighborhood; 3, N5 neighborhood; 4, N6 neighborhood; 5, N7 neighborhood; 6, N8 neighborhood; and 7, N9 neighborhood

To further investigate our data, we applied UMAP (Uniform Manifold Approximation and Projection for Dimension Reduction) [52]. By constructing a low-dimensional embedding of high-dimensional TME and tumor grade data, UMAP revealed patterns and relationships that are challenging to discern in the original high-dimensional space (Fig. 8). Specifically, it identifies clusters of tumors with similar grades and reveals underlying substructures that may be indicative of distinct tumor subtypes. When the UMAP plotting program was applied to raw HLA images from tumors of various grades, it showed normal and tumor-adjacent brain tissue as well separated from the tumor samples. WHO grade 2 tumors are the most spread among others. Tumors of WHO grade 3 and grade 4 form dense and proximate groups in the same location. UMAP plot of phenotypes shows noisy, not very informative patterns. In turn, when phenotypes are grouped into neighborhoods and only after that are applied as input for the UMAP — they reveal a well-defined cluster of the neighborhood N9 — which represents the most abundant feature on the images within glioma dataset Fig. 5, as well as concentrated N2 with the highest significance in comparisons between grades. In this UMAP plot, we can also consider an evaluation of contrastive learning results from the learning and quantifying algorithm protocol.

## Discussion

In this work, we investigated automatic brain tumor grading by using supervised classification and weakly supervised single-cell analysis. The results of the supervised classification demonstrate that simple data augmentation enhanced the network’s performance. As the confusion matrix shows, the model tends to frequently misclassify WHO grade 2 as WHO grade 3 tumors. This is probably related to general difficulties encountered even by experienced histopathologists when attempting to distinguish between grades 2 and 3 of those tumors. The principal distinguishing feature of WHO grade 3 gliomas is the increased mitotic activity and histological anaplasia but the threshold mitotic count for a WHO grade 3 designation has not yet been established. The WHO 2021 classification added molecular markers connected with a poorer prognosis, which is recommended for histological diagnosis in assigning the WHO grade 2 or 3 [53, 54].

We performed single-cell analysis with weakly supervised learning. Differential composition analysis between clusters of TME elements involves identifying differences in the composition of tumor microenvironments between distinct groups of cells or tissue clusters. The goal was to identify specific cellular or molecular features that are associated with different stages of tumor development (and might also correlate with response to treatment). Tumor microenvironment elements include a wide range of cells, such as immune cells, stromal cells, and tumor cells, as well as extracellular matrix molecules and signaling molecules. By analyzing the composition of these elements in different groups of cells or tissue clusters, we can gain insight into the cellular and molecular mechanisms that drive tumor progression.

By using machine learning algorithms such as contrastive learning presented in the previous sections, we can analyze large amounts of brain imaging data more efficiently and accurately. This helps identify subtle patterns of HLA-DR staining that may not be immediately apparent to the human eye, and group together brain regions that exhibit similar patterns of HLA-DR staining. This approach unveils previously unrecognized similarities and differences in the activation of glioma-associated microglia, macrophages, and other immune cells in the brain, providing a more comprehensive understanding of the underlying biological processes that contribute to neurological and psychiatric conditions. The abundance of HLA-expressing myeloid cells and their acquisition of the ameboid phenotype has been demonstrated in glioblastoma [55, 56]. In turn, this information can inform the development of more targeted diagnostic and therapeutic approaches that can improve patient outcomes.

Our approach can be considered an adequate substitute for spatial transcriptomics tools when one wishes to investigate cell populations in TME. There are frameworks available for tissue annotation with pixel-level resolution that integrate histological information with gene expression [57]. In this way, gene expression can be integrated with histological information directly based on the histology image. The method represents a great way to understand the spatial architecture of the TME, however, unlike in our pipeline, it requires a polymerase chain reaction step or even next-generation sequencing (NGS) of the tumor sample.

Our investigation of cell population abundances within and across distinct tumor grades reveals consistent findings that align with previous studies. False positive rates, with satisfying scores when comparing normal brain and low-grade glioma to high-grade glioma and poor results when comparing grades 2 and 3, correspond to the outcomes of the supervised model, as well as to clinical practice. Our evaluation of abundance significance when comparing clusters identified within TME of tumors of various grades did not reveal a significant dissimilarity between WHO grades 2 and 3. We observed, however, that the mean relative abundance of the N9 neighborhood in WHO grade 3 samples is on the order of 0, compared to 0.5 in the case of WHO grade 2. This is consistent with clinical practice, as reliable dissection of WHO grades 2 and 3 tumors is possible with the inclusion of molecular markers.

Gliomas, being intricate tumors with a complex interplay between tumor cells and the immune system, necessitate robust staining methods to unravel their immunological landscape. The choice of tissue staining is far from trivial in this context, as it directly impacts our ability to decipher the intricate immune response within the glioma microenvironment.

HLA-DR staining is a method used in immunohistochemistry to identify the expression of human leukocyte antigen (HLA)-DR, DP, DP, and DQ in tissue samples. HLA-DR is a major histocompatibility complex (MHC) class II antigen that is expressed on the surface of antigen-presenting cells and is critical for the initiation of an immune response. HLA-DR staining can reveal the presence and distribution of myeloid cells such as microglia, monocytes, macrophages, and dendritic cells that are immunosuppressive and tumor supportive [58]. Abundant expression of HLA staining and acquisition of the ameboid phenotype by HLA-expressing cells have been demonstrated in high-grade gliomas [55, 56]. In glioma tissue, malignant gliomas are known to modulate the immune response, leading to an immunosuppressive tumor microenvironment. MHC class II antigens, specifically HLA-DR and DQ, are critical for lymphocyte response and transfection of these genes may boost the immunogenicity of glioma cells [59]. Fan and colleagues [19] consistently found higher mRNA expression of five HLA-DR genes correlates with higher tumor grades, while a higher HLA-DR score, which described the expression of all HLA-DR genes, is also associated with a higher tumor grade. When compared to oligodendroglial tumors, astrocytic tumors — the more aggressive histological subtype of lower-grade gliomas — exhibited a considerably higher HLA-DR score. Tumors with increased HLA-DR expression should have higher immunogenicity, lower aggressiveness, and a better prognosis. However, gliomas with high HLA-DR scores exhibited low immunogenicity, few defined tumor antigens, and more invasive features [60], and patients with high HLA-DR scores had poor survival rates in cases of lower-grade gliomas. This conflicting finding about the relationship between a high HLA-DR score and a poor clinical outcome could result from the immunosuppressive reprogramming of myeloid cells in glioma TME and the complexity of glioma immunogenicity.

Neighborhood N2 abundance shows the highest significance when comparing tumor grade 0 with grade 4, which agrees with the estimation of HLA-DR score measurements performed by Fan et al. [19]. They reported lower HLA-DR mRNA levels in normal brain tissue, low-grade glioma (WHO grade 1, 2), and higher in anaplastic glioma (WHO grade 3), and glioblastoma (WHO grade 4). They also evidenced corresponding differences, including p<0.001 between normal brain tissue and glioblastoma.

Clusters of neighborhoods N2 and N4 extracted from the model (Fig. 4) can represent a pattern of increased HLA-DR staining in WHO grade 4 gliomas indicating accumulation of activated, tumor-supportive myeloid cells within the tumor core and changing the local tissue structure and vascularization. Their increased abundance and activation (reflected by more intensive HLA staining) contribute to a greater complexity of images detected by the protocol. We show that neighborhood N9 occurs more abundantly in WHO grade 2 than in 3.

With the increasing complexity and abundance of imaging data, there is growing optimism that computational methods and advanced image analysis techniques will play a pivotal role in deriving meaningful visual features of tumor clusters. Visual inspection of extracted clusters yields insight into the spatial arrangement, cellular composition, and architectural patterns within the tumor microenvironment, providing a rich source of information for characterizing tumor heterogeneity and progression.

In the pursuit of comprehending histology classification and unraveling the intricate landscape of the tumor microenvironment, we find ourselves confronted with classification criteria which evolve towards more accurate and unambiguous concepts. WHO has adopted “across tumor types” rather than “within a tumor type” grading. Regardless of histological variability, tumors of the same grade have essentially the same clinical outcome. However, the issue of inter- and intra-observer variability remains unresolved. The evaluation of atypia is subjective, while mitotic statistics are dependent on the thoroughness of the examiner as mitoses can accumulate focally or uniformly [53]. The combination of histopathological findings with molecular data allows for a more reliable classification of brain tumors, and the inclusion of unsupervised learning analyses of DNA methylation and next-generation sequencing data has identified distinct subgroups with distinct molecular characteristics [61]. However, the acquisition of DNA methylation and NGS data is expensive and not always affordable for patients; therefore, computational quantification AI-supported protocols dedicated to histology images may contribute to formulating unique descriptors of glioma subtypes complementing diagnosis.

### Study Limitations

The innovative exploration of glioma classification through a combination of weakly supervised learning and single-cell analysis, along with the utilization of tissue microarrays in a general multiclass classification framework, marks a significant contribution to the field of medical image analysis. In spite of that, as with any scientific endeavor, our study is not without its limitations.

- Small imbalanced dataset. The most apparent limitation is the relatively small sample size of the dataset (206 images) and class imbalance. This could impact the generalizability of the results and might limit the robustness of the models trained, particularly in the context of deep learning where larger datasets are often preferred. Strategies to reduce the impact of these limitations included augmentation of underrepresented classes and stratified k-fold cross-validation. In a small dataset, every data point is valuable for training and testing the model. Cross-validation allows for the use of all data points for both training and testing. Each fold gets a chance to be the test set once while the remaining folds form the training set. This approach ensures that all data is used efficiently. Cross-validation mitigates the overfitting and bias risk in the model training by rotating the training and testing sets, providing a more comprehensive view of how the model performs across the entire dataset. Moreover, the performance metrics are averaged over several rounds, offering a more stable and reliable estimate of the model’s performance on unseen data.
- Clinical applicability. The clinical applicability of models generated through the integrated approach shown may be restricted by the complexity of the models, and they may not be practical and accessible for clinical use, at least for the time being.
- Ambiguity in classification boundaries. Histopathologically similar features between different grades can introduce ambiguity in classification boundaries. Subtle morphological differences between WHO grade 2 and 3 gliomas may be challenging for the model to accurately capture, leading to misclassifications. This limitation mainly affects the effectiveness of the supervised deep learning approach — it is one of the reasons why integrating quantifying single-cell analysis into common deep learning classification is advantageous.

## Conclusions

CNN-based diagnostic tools are supervised or weakly supervised deep learning methods that have been effectively applied to a variety of image analyses. They require large labeled datasets, that are not always available in the medical field of rare tumors. We present a new approach dedicated to classifying gliomas, detecting malignant ones, and distinguishing them from normal brain tissues based on images acquired from a single HLA staining of tissues. Given the small sample size and high degree of similarity between WHO grades 2 and 3, this was a particularly challenging task. Even with a limited sample size, the proposed DenseNet121 pretrained HSV selected via architecture search performs relatively well. The application of cell abundance analysis enabled us to retrieve the distinct neighborhood of phenotypes, including N2, which occurs most frequently on TMA core images and exhibits the highest significance when comparing grade 0 and 4 tumors. The second characteristic (differentiating tumor grades) neighborhood of phenotypes is N4, and the second most abundant one is N9. The two phenotypes most frequently present in images of malignant brain tumors are P3 and P4. Our study was designed to combine and compare deep learning whole-slide classification and single-cell analysis in order to elucidate differences between these approaches and investigate how they can complement each other. Our main objective was to investigate pathological aspects of grade progression, particularly focusing on increased myelination. This exploration of hypotheses from existing literature has not been previously validated using automated tools, and our study contributes to bridging this gap in understanding. There is strong hope that automatic grading can become useful during intraoperative settings, as well as to validate the effectiveness of immunotherapies [14]. Future work could provide external validation by evaluating the proposed deep learning model and quantifying protocol on a larger dataset from multiple experiments, as well as integration with clinical data by incorporating information such as patient age, gender, and other relevant medical history, with the potential to improve the model’s accuracy in predicting tumor type and prognosis.

We predict that we may be able to computationally derive features and the corresponding abundances that are characteristic of a particular malignancy grade, to more accurately classify tumors without the need for confirmation via genetic testing.

## New or Breakthrough Work to Be Presented

In this paper, we presented the results of a deep learning multiclass classifier, as well as the discovery and learning protocol of tumor microenvironment elements used in the context of multi-grade gliomas. We obtained our results using single-stained TMA samples. This approach can pave the way to automated tumor grading in clinical settings, saving pathologists’ time. Detecting leukocyte infiltration by immunohistochemical staining of glioma tissue microarrays may improve diagnosis and enable monitoring of the outcome of immunotherapies.

## Data Availability

The image database will be made available once the manuscript is published.

## Code Availability

Code has been made available publically - https://github.com/octpsmon/TME_analysis_protocol_n_glioma_grading.
