# Correction to: Deep Learning Glioma Grading with the Tumor Microenvironment Analysis Protocol for Comprehensive Learning, Discovering, and Quantifying Microenvironmental Features

**DOI:** 10.1007/s10278-024-01160-4

**Published:** 2024-06-12

**Authors:** M. Pytlarz, K. Wojnicki, P. Pilanc, B. Kaminska, A. Crimi

**Affiliations:** 1 Sano - Centre for Computational Personalised Medicine, Czarnowiejska 36, Kraków, 30-054 Poland; 2 Nencki Institute of Experimental Biology of the Polish Academy of Sciences, 3 Pasteur Street, Warsaw, 02-093 Poland

**Correction to: Journal of Imaging Informatics in Medicine** 10.1007/s10278-024-01008-x

We would like to update the “Availability of Data and Materials” with the link to the dataset.

The dataset, “Glioma—Human Leukocyte Antigen Tissue Microarray. Data from: Deep Learning Glioma Grading with the Tumor Microenvironment Analysis Protocol for Comprehensive Learning, Discovering, and Quantifying Microenvironmental Features,” is now available in the Zenodo repository and can be accessed at https://zenodo.org/records/10807474.

In the section “Conclusions”:

- In the sentence, “Our main objective was to investigate pathological aspects of grade progression, particularly focusing on increased myelination,” the term “increased myelination” should be replaced with “increased myeloid cell accumulation” as we discuss myeloid cell accumulation throughout the article.

